# Applying the New Inflammation Criterion Impairs GLIM Validity in Hospitalized Patients with Acute Medical Conditions

**DOI:** 10.3390/nu18030462

**Published:** 2026-01-30

**Authors:** Laia Fontané, Maria Helena Reig, Míriam Herranz, Maria Antonia Roig, Altea Pérez, Juan José Chillarón, Araceli Estepa, Silvia Toro, Humberto Navarro, Gemma Llauradó, Juan Pedro-Botet, David Benaiges

**Affiliations:** 1 Department of Endocrinology and Nutrition, Consorci Sanitari Alt Penedès-Garraf, Espirall, 61, 08720 Vilafranca del Penedès, Spain; lfontane@hmar.cat (L.F.); hreig@csapg.cat (M.H.R.); mherranz@csapg.cat (M.H.); maroig@csapg.cat (M.A.R.); jjchillaron@csapg.cat (J.J.C.); aestepa@csapg.cat (A.E.); storo@csapg.cat (S.T.); hnavarro@csapg.cat (H.N.); 2 Department of Endocrinology and Nutrition, Hospital del Mar, Passeig Marítim, 25-29, 08003 Barcelona, Spain; apereze@csapg.cat (A.P.); gllaurado@hmar.cat (G.L.); jpedrobotet@hmar.cat (J.P.-B.); 3 Medical and Life Sciences (MELIS) Department, Universitat Pompeu Fabra, Plaça de la Mercè, 10-12, 08002 Barcelona, Spain; 4 Institut Hospital del Mar d’Investigacions Mèdiques (IMIM), Dr. Aiguader, 80, 08003 Barcelona, Spain; 5 Center for Biomedical Research on Diabetes and Associated Metabolic Diseases (CIBERDEM), Instituto de Salud Carlos III (ISCIII), 28029 Barcelona, Spain; 6 Centro de Investigación Biomédica en Red de la Fisiopatología de la Obesidad y Nutrición, Monforte de Lemos Avenue, 3-5, Pavilion 11, Floor 0, 28029 Madrid, Spain

**Keywords:** global leadership initiative on malnutrition, hospitalized patients, inflammation, malnutrition, nutrition assessment

## Abstract

**Background/Objectives**: The Global Leadership Initiative on Malnutrition (GLIM) recently updated its inflammation criterion through a Delphi consensus to standardize its assessment. This study aimed to assess the impact of these new recommendations on the concurrent and predictive validity of the GLIM criteria in hospitalized medical patients. **Methods**: This post hoc analysis re-evaluated a previously published cohort of 119 hospitalized patients with acute medical conditions, originally assessed using the GLIM criteria and the Subjective Global Assessment (SGA) as the reference standard. Inflammation was redefined according to the 2024 GLIM Delphi consensus, and the concurrent and predictive validity of the modified GLIM criteria (GLIM-I) were examined. Receiver operating characteristic (ROC) curves were used to compare the discriminative ability of SGA, original GLIM, and GLIM-I to predict prolonged hospital stay. **Results**: With the updated inflammation definition, all patients met the etiologic criterion, increasing malnutrition prevalence from 41.7% to 52.2%. GLIM-I showed a sensitivity of 78.0% and specificity of 67.7% versus SGA, not reaching the predefined ≥80% threshold for concurrent validity. Predictive validity was maintained (adjusted odds ratio (OR) = 3.40; 95% CI: 1.31–8.83). SGA achieved the highest discriminative ability (area under the curve (AUC) = 0.783; 95% CI: 0.693–0.874), significantly outperforming the original GLIM (AUC = 0.723; 95% CI: 0.616–0.830; *p* = 0.049). GLIM-I showed similar performance (AUC = 0.731; 95% CI: 0.620–0.843; *p* = 0.727). **Conclusions**: SGA should continue to be considered the method of choice for nutritional diagnosis in hospitalized medical patients. Further research is needed to determine how the new inflammation criteria influence the validity of the GLIM framework in other clinical contexts before their widespread implementation.

## 1. Introduction

Disease-related malnutrition is highly prevalent in hospitalized patients and is linked to increased morbidity, mortality, and healthcare costs [1]. Therefore, accurate diagnosis is essential to guide timely nutritional interventions and improve outcomes. The Subjective Global Assessment (SGA) has traditionally been the reference tool due to its simplicity, feasibility, and predictive value [1]. In 2018, the Global Leadership Initiative on Malnutrition (GLIM) proposed a standardized diagnostic framework endorsed by major clinical nutrition societies [2]. These criteria, conceived to reduce the subjectivity of the SGA and provide a more objective assessment of malnutrition, were intended to become the new gold standard. To achieve this, however, their validity had to be demonstrated. For this reason, the GLIM group proposed a specific validation methodology, setting thresholds of ≥80% sensitivity and specificity for concurrent validity, and an odds ratio (OR) ≥ 2 for predictive validity based on clinical outcomes such as prolonged hospital stay [3].

In 2023, our group published a prospective validation study evaluating the performance of the GLIM criteria in patients hospitalized for acute medical conditions, using the SGA as the reference standard [4]. Among the 119 participants, GLIM showed a sensitivity of 78.0% (95% CI: 64.0–88.5) and a specificity of 86.2% (95% CI: 75.3–93.5), thus not reaching the predefined threshold for concurrent validity. Nevertheless, predictive validity was confirmed, as patients identified as malnourished by GLIM had a higher risk of prolonged hospital stay (adjusted OR: 2.98; 95% CI: 1.21–7.60).

Since their publication, the GLIM criteria have been widely accepted and are being progressively implemented in clinical practice. According to an international survey conducted among more than 1500 professionals, 25% reported that the GLIM criteria had already been implemented in their practice, while another 20% indicated that implementation was in progress [5]. Moreover, five years after the original proposal, the GLIM consensus group published an updated statement summarizing the progress made in GLIM criteria implementation and validation [6]. Drawing on the main systematic reviews and meta-analyses available, the authors concluded that the GLIM criteria show good predictive validity and acceptable concurrent validity, supporting their broader clinical use. However, they also acknowledged the substantial variability observed across studies. One of the main contributors to this heterogeneity was the absence of standardized guidance for assessing the etiologic criterion of inflammation. To address this gap, in 2024, the GLIM working group released a modified Delphi consensus providing specific recommendations for its evaluation [7]. In our previous study, inflammation had been defined according to *C*-reactive protein (CRP) levels, which differs from the newly proposed approach. Consequently, we reanalyzed the concurrent and predictive validity of our cohort applying these updated recommendations.

## 2. Materials and Methods

**Figure 1:**
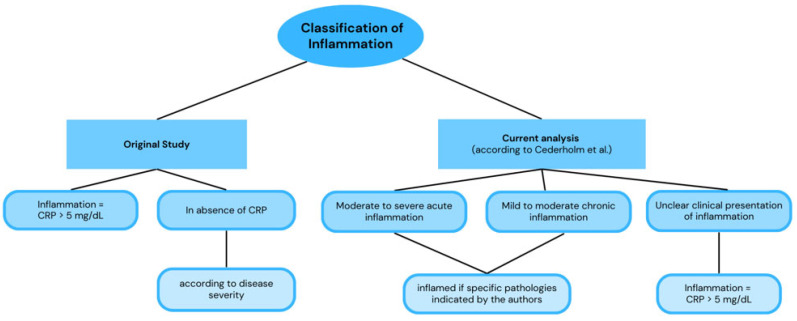
Classification of inflammation. Abbreviations: CRP = *C*-reactive protein. Unclear clinical presentation of inflammation, e.g., psychiatric diagnoses like anorexia nervosa and depression; select malabsorptive, obstructive, or dysmotility conditions like esophageal stricture, anatomic short bowel syndrome; intestinal pseudo-obstruction; neurological conditions like dysphagia after cerebrovascular accident. Pathologies included and considered as inflammatory: chronic diseases complicated by acute moderate exacerbations or acute new presentations with moderate inflammation associated with, e.g., Crohn’s disease, chronic obstructive pulmonary disease (COPD), pancreatitis, diabetes, infections, wounds, cancer, congestive heart failure, cystic fibrosis, and others. Cederholm et al. [7].

The present study was a post hoc analysis of a prospective cohort conducted in patients hospitalized for acute medical conditions at the Consorci Sanitari de l’Alt Penedès-Garraf between April and October 2022. In the original study, the validity of the nutritional diagnosis using the GLIM criteria was assessed, with the SGA serving as the reference standard [4]. The detailed methodology has been previously described [4]. In the current analysis, the only methodological change concerned the definition of inflammation, which was re-evaluated according to the consensus guidance proposed by Cederholm et al. [7]. Figure 1 illustrates how inflammation was defined in both the original and the current analyses. In the original analysis, inflammation was primarily determined using CRP levels, whereas in the present reanalysis, inflammation was defined mainly on clinical criteria, based on the presence of diseases or conditions associated with inflammatory activity, in accordance with the Delphi consensus (see Figure 1 legend).

Statistical analyses were performed following the methodology described in our previous study [4] and in accordance with the validation framework proposed by the GLIM authors [3]. In this reanalysis, the concurrent validity of the modified GLIM criteria incorporating the new inflammation definition (hereafter referred to as GLIM-I) was evaluated. Predictive validity was then assessed, as in our previous analysis with SGA and the original GLIM criteria, by calculating adjusted ORs for prolonged hospital stay (>10 days). The analyses were adjusted for sex, age, Charlson comorbidity index, and Barthel index. In addition, receiver operating characteristic (ROC) curves were developed to assess the discriminative ability of each method (SGA, original GLIM, and GLIM-I). Subsequently, the equality between the different ROC curve areas obtained was tested using the DeLong test.

## 3. Results

As previously reported, GLIM criteria could be applied to 115 of the 119 patients included in the original study cohort. The characteristics of the full cohort, including comparisons between malnourished and non-malnourished patients according to the original GLIM definition, have been thoroughly detailed in our prior publication [4].

When applying the updated definition of inflammation, 100% of patients met the etiologic criterion, as all were classified as inflamed. Consequently, the number of patients diagnosed with malnutrition according to GLIM increased from 48 (41.7%) in the initial analysis to 60 (52.2%) in this reassessment. Table 1 shows the characteristics of patients classified as malnourished or well-nourished based on the updated GLIM-I criteria. Notably, unlike what was observed in our previous publication, phenotypic variables such as calf circumference and reduced muscle mass no longer show statistically significant differences between malnourished and non-malnourished patients. A similar pattern is observed for Body Mass Index; although the absolute BMI value remains significantly different between groups, the low-BMI cutoff belonging to the phenotypic criterion also loses its statistically significant discriminatory capacity.

The main findings from both the original study and the present reanalysis are summarized in Table 2. When applying the modified criteria (GLIM-I), the concurrent validity threshold was still not achieved, as sensitivity and/or specificity remained below the predefined 80% cutoff.

**Figure 2:**
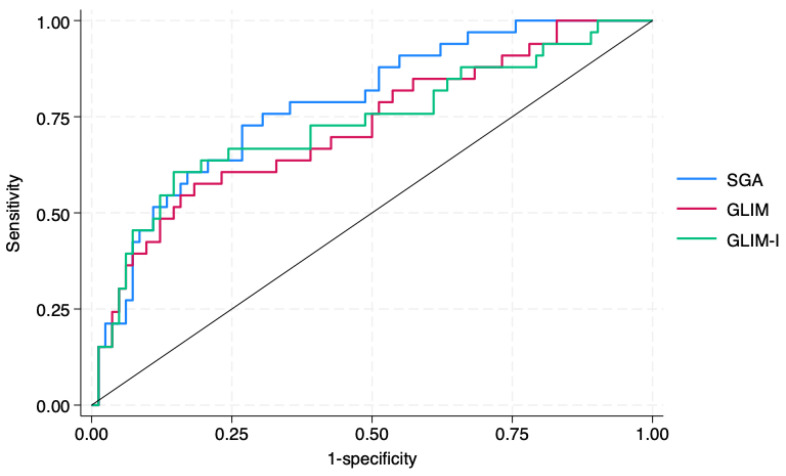
Adjusted ROC curves comparing the predictive performance of SGA and GLIM. Abbreviations: GLIM = Global Leadership Initiative on Malnutrition; SGA = Subjective Global Assessment.

As shown in Table 2, the GLIM-I model met the predefined criterion for predictive validity, as malnourished patients presented a significantly higher risk of prolonged hospital stay (>10 days). To further compare the predictive performance of the different diagnostic approaches, Figure 2 displays the adjusted ROC curves for SGA, the original GLIM, and GLIM-I. The SGA demonstrated the highest discriminative ability, with an area under the curve (AUC) of 0.783 (95% CI: 0.693–0.874), which was significantly greater than that of the original GLIM criteria (AUC = 0.723; 95% CI: 0.616–0.830; *p* = 0.049 by DeLong’s test for AUC comparison). The GLIM-I model reached an AUC of 0.731 (95% CI: 0.620–0.843), not significantly different from the original GLIM model (*p* = 0.727).

## 4. Discussion

The present study reveals key weaknesses in the GLIM framework, mainly related to two factors that directly affect its validity and explain the variability across studies. The first one is the limited applicability of the newly proposed definitions for inflammation, and the second one is the specific challenges associated with their use in hospitalized patients.

Inflammatory biomarkers have well-known limitations and are susceptible to confounding by comorbidities and age-related factors, which are particularly prevalent in hospitalized patients and may lead to false-positive classifications [8]. To address these issues, the newly proposed GLIM inflammation criterion adopts a more clinically oriented approach, in which inflammatory biomarkers such as CRP play a secondary role. This modification is conceptually sound from a nutritional standpoint and was endorsed by 99% agreement in the recent Delphi consensus. However, in our cohort of patients hospitalized for acute medical conditions, its application compromised the validity of the GLIM framework. The new definition classified all patients as inflamed, resulting in an approximate 25% increase in malnutrition diagnoses. This led to a higher rate of false positives, lowering specificity and preventing the model from reaching the predefined ≥80% threshold for concurrent validity. In parallel, when all patients automatically fulfill one etiologic criterion, the diagnostic contribution of reduced intake or absorption becomes negligible, and any single phenotypic alteration leads to a diagnosis of malnutrition. This situation likely reflects a loss of diagnostic precision, as suggested by the absence of significant differences in the phenotypic criteria of low body mass index and reduced muscle mass between malnourished and well-nourished patients, in contrast to the clear distinctions observed in the original analysis [4].

To date, only one validation study has implemented the new inflammation guidelines. Wu et al. [9] assessed 140 hospitalized patients with nasopharyngeal carcinoma at admission and discharge, reporting poor concurrent validity of GLIM when compared with SGA, with sensitivities and specificities of 68.8% and 81.5% at admission, and 99% and 10.9% at discharge. Similarly, Liu et al. [10], although not primarily designed as a validation study, found that applying the new inflammation standards led to an overestimation of malnutrition, as all patients in their cohort met the etiologic criterion.

Beyond the definition of inflammation, the hospital setting itself poses specific challenges for the application of the GLIM criteria. Results of most studies conducted in this context concurred with our findings, failing to meet the predefined thresholds for concomitant validity [11,12,13,14,15]. Across these studies, diagnostic performance was highly variable, with sensitivity values ranging from 43% to 96%, specificity values between 15% and 89.7%, and none achieving both sensitivity and specificity above the predefined ≥80% validation threshold. Moreover, in the systematic review by Alves et al. [16], for instance, only one out of seven studies comparing GLIM with validated diagnostic tools achieved the required criteria for concurrent validity.

From a conceptual and nutritional viewpoint, the GLIM framework could theoretically be considered a more objective and comprehensive tool for diagnosing malnutrition than the SGA. However, our findings do not support this hypothesis in acutely hospitalized medical patients within the context of a single-center cohort and may not be generalizable to other clinical settings. Although GLIM-I formally met the predefined criterion for predictive validity, this alone is insufficient to support its clinical implementation in this setting. First, GLIM-I failed to achieve concurrent validity, limiting its diagnostic accuracy at the individual patient level. Second, its predictive performance was significantly inferior to that of the SGA, as demonstrated by a lower AUC for prolonged hospital stay. Beyond these results, our previous analysis also showed that SGA is a more practical tool, as it can be completed in less time and requires fewer resources than GLIM—an important consideration in hospital settings where qualified nutrition professionals are often limited [4].

In our study, male sex was significantly associated with a higher prevalence of malnutrition. This finding is consistent with previous reports and may be explained, at least in part, by the higher prevalence of certain chronic diseases (such as diabetes mellitus, chronic bronchitis, and emphysema), poorer dietary habits, and greater alcohol and tobacco consumption observed among men in our clinical setting, all of which may increase the risk of malnutrition [17].

This study has several limitations that should be acknowledged. First, it is a post hoc reanalysis of a previously published cohort, which may limit the scope of the conclusions. Second, the relatively small sample size restricts the statistical power and may limit external validity. Third, as the study was conducted in a single cohort of patients hospitalized for acute medical conditions, the findings may not be generalizable to other clinical settings or patient populations. Finally, although length of hospital stay was analyzed as a measure of predictive validity and adjusted for age, sex, Barthel Index, and Charlson Comorbidity Index, it should be acknowledged that length of hospital stay is a multifactorial outcome and remains susceptible to residual confounding despite adjustment for relevant clinical variables.

Together, these findings suggest that the observed loss of validity is primarily related to the application of the updated inflammation criterion in the specific context of acute hospitalization, rather than representing an intrinsic limitation of the GLIM framework itself. GLIM was originally conceived as a flexible diagnostic approach applicable across a wide range of clinical scenarios, many of which are characterized by heterogeneous or intermittent inflammatory burden. In contrast, acute medical hospitalization represents a setting in which inflammation is frequently present, and in our cohort, the updated definition resulted in near-universal fulfillment of the etiologic inflammation criterion. This situation challenges the discriminative capacity of GLIM in this specific context but does not necessarily extend to other clinical environments, such as ambulatory oncology care, long-term care facilities, or rehabilitation settings, where inflammation is not systematically present. Importantly, our findings highlight the need to specifically evaluate how the new inflammation criteria affect the validity of GLIM across different clinical settings before their broad implementation. In this context, improving the specificity of GLIM in hospitalized patients may require a more nuanced definition of inflammation that better reflects the clinical reality of acute illness. Approaches incorporating degrees of inflammation, rather than a purely binary classification, and combining clinical assessment with selected biochemical markers (e.g., CRP), may help reduce false-positive diagnoses. Further studies are needed to determine whether such refinements could improve the diagnostic performance of GLIM in hospitalized medical patients.

## 5. Conclusions

SGA should continue to be considered the method of choice for nutritional diagnosis in hospitalized medical patients, based on findings from this single-center cohort study. At the same time, further research is needed to determine how the new inflammation criteria influence the validity of the GLIM framework in other clinical contexts before their widespread implementation.

## Figures and Tables

**Table 1:** Characteristics of patients with and without malnutrition according to GLIM criteria, using the new inflammation definition from the modified Delphi consensus [7].

Variable	Well Nourished (*n* = 55)	Malnourished (*n* = 60)	*p* Value
Sociodemographic data			
Age (years)	66.2 ± 14.7	64.5 ± 15.5	0.274
Women (%)	61.8	40.0	0.016
Caucasian (%)	100	95	0.244
Medical data			
Charlson index	1.63 ± 1.7	2.4 ± 2.6	0.028
Barthel index	81.1 ± 28.2	85.3 ± 20.8	0.179
Analytical parameters			
Albumin (mg/dL)	3.25 ± 0.47	2.76 ± 0.59	<0.001
CRP ^1^ (mg/dL)	1.27 ± 1.24	24.1 ± 41	<0.001
Nutritional characteristics			
Current weight (kg)	72.6 ± 14.8	70 ± 16.1	0.200
Body mass index (kg/m^2^)	27.4 ± 5.9	25.3 ± 5.7	0.037
Weight loss (kg)	0.42 ± 4.62	5.3 ± 6.4	<0.001
Circumference of calf (cm)	33.6 ± 5	32.5 ± 3.1	0.085
Presence of phenotypic criteria			
Altered weight loss (*n*, %)	14 (25.5%)	34 (56.7)	0.003
Nonmeasurable weight loss (*n*, %)	8 (14.5%)	6 (10%)	
Low Body Mass Index (*n*, %)	4 (7.3%)	10 (16.7%)	0.306
Nonassessable Body Mass Index (*n*, %)	8 (14.5%)	8 (13.3%)	
Reduced muscle mass (*n*, %)	23 (41.8%)	30 (50%)	0.550
Nonassessable reduced muscle mass (*n*, %)	1 (1.8%)	2 (3.3%)	
Presence of etiological criteria			
Inflammation (*n*, %)	55 (100%)	60 (100%)	1
Dietary intake or reduced absorption (*n*, %)	0	34 (56.7%)	<0.001

^1^ CRP = *C*-reactive protein.

**Table 2:** Concurrent and predictive validity of the original study and the current analysis.

	Original Study [4]	Current Analysis
Concurrent validity (GLIM ^1^ vs. SGA ^2^)		
Sensitivity (%, 95% CI ^3^)	78.0 (64.0–88.5)	78.0 (66.5–89.5)
Specificity (%, 95% CI ^3^)	86.2 (75.3–93.5)	67.7 (56.3–79)
Positive predictive value (%, 95% CI ^3^)	83.6 (75.0–89.7)	65 (52.3–77.1)
Negative predictive value (%, 95% CI ^3^)	81.3 (69.9–89.0)	80 (69.4–90.6)
AUC ^4^ ROC ^5^ (95% CI)	0.82 (0.74–0.90)	0.73 (0.63–0.82)
Weighted Kappa (95% CI)	0.64 (0.50–0.79)	0.44 (0.29–0.61)
Predictive validity (hospital stay > 10 days)		
OR ^6^ GLIM ^1^	2.98 (1.21–7.60)	3.40 (1.31–8.83)
OR ^6^ SGA ^2^	6.16 (2.42–7.08)	6.16 (2.42–7.08)

^1^ GLIM = Global Leadership Initiative on Malnutrition; ^2^ SGA = Subjective Global Assessment; ^3^ CI = confidence interval; ^4^ AUC = area under the curve; ^5^ ROC = receiver operating characteristic; ^6^ OR = odds ratio.

## Data Availability

Sensitive data cannot be publicly available, but the data can be made available by an appropriate request to the corresponding author.

## Abbreviations

The following abbreviations are used in this manuscript:

GLIM	Global Leadership Initiative on Malnutrition
SGA	Subjective Global Assessment
GLIM-I	Modified GLIM criteria
ROC	Receiver operating characteristic
OR	Odds ratio
AUC	Area under the curve
CRP	*C*-reactive protein
